# Public health surveillance perspectives from provincial COVID-19 experiences, South Africa 2021

**DOI:** 10.4102/jamba.v16i1.1625

**Published:** 2024-10-17

**Authors:** Ruvimbo Chingonzoh, Yvonne Gixela, Bontle Motloung, Nosiphiwo Mgobo, Zonwabele Merile, Thomas Dlamini

**Affiliations:** 1 Division of Public Health Surveillance and Response, National Institute for Communicable Diseases, Johannesburg, South Africa; 2 Epidemiology and Research Unit, Eastern Cape Provincial Department of Health, Bhisho, South Africa; 3 World Health Organization, Pretoria, South Africa

**Keywords:** COVID-19 surveillance, surveillance system review, public health surveillance, public health emergencies, integrated surveillance, sub-national

## Abstract

Previous pandemics, recent outbreaks, and imminent public health events are a clarion call for functional public health surveillance systems that timeously detect public health events, guide interventions, and inform public health policy. We reviewed the Eastern Cape Provincial coronavirus disease 2019 (COVID-19) surveillance approach to determine best practices and opportunities to strengthen public health surveillance. We conducted a document review of COVID-19 surveillance reports, tools and guidelines prepared between March 2020 and November 2021. Iterative content and thematic analysis were applied to identify strengths and shortcomings of provincial COVID-19 surveillance. Strengths and shortcomings of the provincial COVID-19 surveillance process, and human, technical, and technological resources for surveillance were described. The existence of local surveillance networks, local availability of national-level surveillance guidelines, the ability to describe and track COVID-19 epidemiology, and provincial access to a national, web-based centralised COVID-19 surveillance data system were strengths identified. Shortcomings included poor data quality, data disharmony between sub-national reporting levels, under-resourced surveillance capacity at district level, and suboptimal use of the routine surveillance system for COVID-19 surveillance. The review determined the need for a web-based, integrated surveillance system that was agile in meeting evolving surveillance needs and accessible at all health reporting levels for response and decision-making.

**Contribution:**

The review identified opportunities to advance the existing routine public health surveillance system and improve public health surveillance and response. This qualitative review articulates local knowledge that should be translated into strategies and actions to bolster public health preparedness.

## Introduction

Public health surveillance is an ongoing systematic process where health data are collected, collated, analysed, interpreted, and disseminated to those who need to know and act (Adebisi, Rabe & Lucero-Prisno Iii 2021; Khamis Ibrahim 2020; Nsubuga et al. 2006). The broad objective of surveillance is to provide health information to guide decision-making and direct public health interventions (Nsubuga et al. 2006). Surveillance systems are set up to, among others, timeously detect public health threats, understand population-level disease burden, determine public health priorities, evaluate health programmes and existing interventions, and provide a basis for epidemiology-related research (Gold et al. 2021; Groseclose & Buckeridge 2017; Nsubuga et al. 2006; WHO 2014). Public health surveillance is a cornerstone for early warning alert and response to public health events (WHO 2014).

The coronavirus disease 2019 (COVID-19) pandemic emphasised the importance of public health surveillance in understanding the burden and risk factors of COVID-19 (Adebisi et al. 2021; Judson et al. 2022; Ricks et al. 2022). Surveillance of COVID-19 facilitated case detection and management; outbreak identification and containment; development, implementation, and review of targeted control measures; monitoring of epidemiological trends, including COVID-19 hospitalisation and mortality; and tracking the evolution of severe acute respiratory syndrome coronavirus 2 (SARS-CoV-2) (Gold et al. 2021; Jassat et al. 2021; National Institute for Communicable Diseases [NICD] 2023a; Tessema et al. 2020).

The COVID-19 pandemic highlighted and amplified health system weaknesses (Morgan et al. 2021; Sagan et al. 2021; Saikat et al. 2023), including health system gaps such as inadequate diagnostic capability and fragmented data systems, which contributed to suboptimal capability for case and cluster detection (Adebisi et al. 2021; Arvisais-Anhalt et al. 2021; Morgan et al. 2021). Even before the COVID-19 pandemic, public health surveillance within the African region, including implementing the Integrated Disease Surveillance and Response (IDSR) strategy, has been beset with challenges. Such challenges include poor data management, parallel data reporting systems, limited funding, and human resource challenges such as lack of trained surveillance workforce, and surveillance posing an additional burden on health facility staff (Adebisi et al. 2021; Nsubunga et al. 2010; Wolfe et al. 2021).

Strengthening public health surveillance not only facilitates local, regional, and global public health preparedness and response, but also ensures that public health decision-making is based on real-time, accurate epidemiologic and clinical surveillance data (IFRCS 2020; Morgan et al. 2021; Rogers et al. 2020). Strategies to strengthen public health surveillance include using digital technology and adequately financing public health surveillance (Morgan et al. 2021).

Previous pandemics, recent outbreaks, and imminent public health events are a clarion call for functional public health surveillance systems that timeously detect public health events, guide public health interventions, and inform public health policy (Adebisi et al. 2021; Khamis Ibrahim 2020; Nsubunga et al. 2010). A recent editorial on health systems recovery in the context of COVID-19 highlights the need for an active approach to sustain and develop strategies that serve well rather than passively reverting to pre-existing health system baselines (Saikat et al. 2023). For the Eastern Cape Department of Health to translate lessons learned during COVID-19 response into actions that strengthen routine public health surveillance and surveillance during public health emergencies, the provincial health authority has to understand what worked well and what did not. Reviewing the provincial COVID-19 surveillance process provides evidence to inform the development and implementation of strategies to either sustain COVID-19 surveillance strengths or address COVID-19 surveillance shortcomings. We undertook a province-level COVID-19 surveillance document review to describe the provincial COVID-19 surveillance process between March 2020 and February 2021, establish province-level COVID-19 surveillance best practices and province-level COVID-19 surveillance shortcomings, and provide recommendations to strengthen the provincial public health surveillance system.

## Research method and design

### Research setting

We reviewed the Eastern Cape COVID-19 surveillance approach through qualitative analysis of provincial COVID-19 surveillance documents.

### Area description

The Eastern Cape Province, the fourth most populous province of nine South African Provinces, accounts for 11.0% of the South African population (Statistics South Africa 2022b). The province is predominantly rural and comprises six district and two metropolitan municipalities (Statistics South Africa 2018). The province has the third lowest medical aid coverage, with 10.6% of the provincial population covered by a medical aid scheme (Statistics South Africa 2022a). In January 2023, the province accounted for the sixth highest COVID-19 cumulative risk (5487 cases per 100 000 persons) (NICD 2023a), and the highest in-hospital COVID-19 case fatality rate (27.2%) (NICD 2022) in the country. The province conducts routine public health surveillance via the Notifiable Medical Conditions Surveillance System (NMCSS). This national-level, passive, indicator-based surveillance system includes an electronic reporting platform and a paper-based platform, and it incorporates data from the public and private health sectors. By law, clinicians who diagnose a notifiable medical condition must report it via the NMCSS, as stipulated in the national notifiable medical conditions regulations of South Africa (NICD 2021).

### Data sources

Coronavirus disease 2019 surveillance-related texts were sourced from the Epidemiology and Research Unit of the Eastern Cape Department of Health. Institutional records sourced included provincial surveillance guidelines, surveillance tools and epidemiological reports prepared between 01 March 2020 and 30 November 2021. Provincial COVID-19 surveillance documents selected included provincial surveillance documents crafted to inform the provincial COVID-19 surveillance approach. Provincial COVID-19 surveillance reports included epidemiological reports, outbreak reports, and other ad hoc surveillance reports. Such reports reflected the surveillance processes and related strengths and shortcomings of COVID-19 surveillance in the province.

The province-level COVID-19 surveillance document review was augmented by reviewing and triangulating with findings of the provincial COVID-19 Intra Action Review (IAR) conducted on 29 and 30 September 2020. The provincial COVID-19 IAR adopted a World Health Organization (WHO) methodology (WHO 2021), whereby key informant accounts were collected through structured facilitator-led discussions. Key informants of the IAR were from provincial, district and local municipality levels, and included COVID-19 response stream leads, teams and representatives from COVID-19 response pillars that were under review. Intra Action Review participant accounts were recorded and analysed for remediation of the then ongoing response. Table 1 summarises COVID-19 surveillance documents that we retained and reviewed.

**TABLE 1:** A summary of COVID-19 surveillance documents included for document review and analysis: Eastern Cape, 2020–2021.

Document name	Type	Author(s)
Provincial epidemiological report for SARS-Cov-2	Situational Reports	ECDoH COVID-19 Epidemiology and Surveillance Team
District-level COVID-19 reports	Situational Reports	ECDoH COVID-19 District Response Teams
Guiding document for COVID-19 surveillance information flow	Provincial Guideline	ECDoH COVID-19 Epidemiology and Surveillance Team
Eastern Cape province COVID-19 case reporting process	Standard Operating Procedure	ECDoH COVID-19 Epidemiology and Surveillance Team
Mapping COVID-19 data sources	Ad hoc Report	ECDoH COVID-19 Epidemiology and Surveillance Team
COVID-19 outbreak reports	Ad hoc Reports	Provincial and District COVID-19 Response Teams
Eastern Cape intra action review report: 29–30 September 2020	Report	ECDoH COVID-19 Intra Action Review Team

### Document review

#### Document screening

Documents were screened to ensure authenticity and relevance to COVID-19 surveillance at a provincial level. Coronavirus disease 2019 surveillance documents were included based on the document objective (intended to inform or report COVID-19 surveillance), authorship, target audience, and availability of original publication and subsequent revisions. The document selection criteria, implemented to manage selection bias, was applied to all surveillance-related documents sourced from the Eastern Cape Department of Health. Integration of information from diverse data sources, including the IAR report, mitigated potential bias related to using of limited data sources.

#### Analysis

**FIGURE 1:**
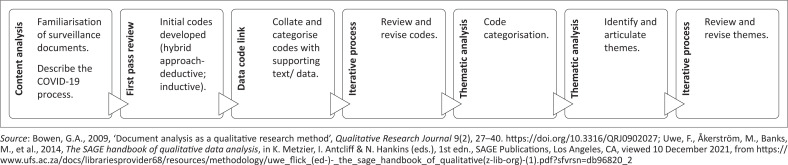
Stepwise approach to content and thematic review of provincial COVID-19 surveillance documents; Eastern Cape, 2020–2021.

We applied a combination of content and thematic analysis with manual coding of provincial COVID-19 surveillance documents. Qualitative analysis of provincial COVID-19 surveillance documents employed both deductive (researcher-based codes) and inductive coding (codes derived from document review), as summarised in Figure 1.

#### Description of the provincial COVID-19 surveillance process

We conducted content analysis to describe the provincial COVID-19 surveillance process. We applied process coding to identify text related to COVID-19 surveillance actions and activities, and text related to the sequence of COVID-19 surveillance actions. We applied thematic analysis using structural coding to identify COVID-19 surveillance themes and concepts related to COVID-19 information relay, COVID-19 surveillance data sources, and COVID-19 surveillance outputs. Structural codes were applied to identify COVID-19 reporting practices, information reporting flow, and the sequence of actions and activities related to provincial COVID-19 surveillance. Descriptive coding was used to identify additional themes related to the COVID-19 surveillance process. The combination of thematic and content analysis responded to the first research objective, which described the provincial COVID-19 surveillance process.

#### Establishing best practices and shortcomings of provincial COVID-19 surveillance

We applied thematic analysis to establish the strengths and shortcomings of provincial COVID-19 surveillance. We crafted structural codes related to the research questions. The structural codes were related to questions on (1) provincial COVID-19 surveillance strengths and (2) provincial COVID-19 surveillance shortcomings. Wherever emerging topics were identified, we inductively created descriptive codes from the emerging topics. Code categories were constructed and organised thematically based on common threads and emerging COVID-19 surveillance topics. We applied a combination of iterative qualitative analysis and hybrid coding to establish best practices and shortcomings of provincial COVID-19 surveillance.

The external validity of the review was established by presenting the document review findings to local public health specialists involved in provincial and national COVID-19 surveillance. Comments and inputs were addressed and considered before finalising the review.

### Ethical considerations

This document review was conducted within the ethical clearance (certificate M210752) granted by the Human Research Ethics Committee (Medical) of the University of the Witwatersrand to the NICD operational research project titled: *Essential communicable disease surveillance and outbreak investigation activities of the National Institute for Communicable Diseases (NICD)*. Provincial approval was granted by the Eastern Cape Provincial Health Research Committee Secretariat (reference number EC_202202_010). This qualitative review of COVID-19 surveillance did not include studies involving human participants performed by the authors.

## Results

The document review identified four themes, which were as follows: (1) provincial COVID-19 surveillance process, and (2) technical, (3) human, and (4) technological resources for surveillance. The provincial COVID-19 surveillance process, which was the first theme, comprised of sub-themes related to COVID-19 surveillance activities, information pathways, use of COVID-19 surveillance data, and strengths and shortcomings of the surveillance process.

### Description of provincial COVID-19 surveillance process

#### Surveillance process and information pathways

**FIGURE 2:**
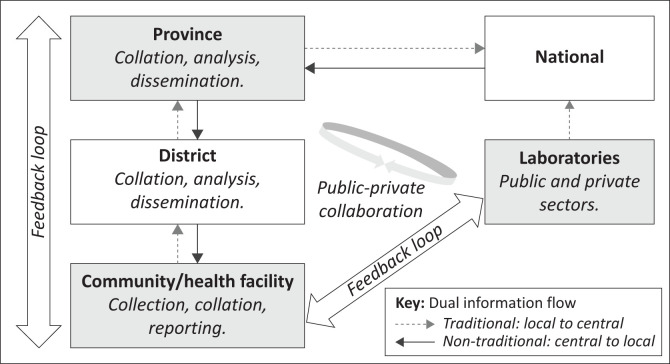
The COVID-19 surveillance information pathways, Eastern Cape, 2020–2021.

The review mapped the main COVID-19 surveillance activities at different health reporting levels. At community and health facility levels, surveillance activities were related to COVID-19 screening, specimen collection, case follow-up and management, close contact identification and tracking, and management and reporting of COVID-19 data. Surveillance activities at the health laboratory level, included laboratory testing, data collation, and reporting. Surveillance activities at the local municipality, district and provincial levels included collating and analysing data and disseminating surveillance information to stakeholders. Figure 2 represents a simplified schematic of the provincial COVID-19 surveillance process and information pathways.

Coronavirus disease 2019 surveillance information relay comprised of traditional and non-traditional information flow. Traditional information flow maintained the routine, hierarchical health information flow where data were recorded at the local level (community and health facility) and transmitted upwards to intermediate (district and provincial) and central (national) levels. At the health laboratory level, there was multilevel flow of COVID-19 laboratory data to patients, public and private healthcare providers, and centrally to the national level. Provision of COVID-19 data to local health reporting levels facilitated local public health response.

The review determined that centralised collation and reporting of COVID-19 case data introduced non-traditional COVID-19 information flow. Non-traditional information flow involved downward reporting of COVID-19 test and confirmed case data from the national level to the provincial level and from the provincial level to the district and subsequently to local levels. Coronavirus disease 2019 test and confirmed case data were collated and managed at the national level and accessed at the provincial level. At the provincial level, further processing of case data, including geolocation of COVID-19 cases, was conducted before case data were disseminated to districts. At the district level, cases not already detected via existing local surveillance networks, such as local laboratory networks and general practitioner networks, were identified and forwarded to local municipalities and respective response teams. This information flow was alluded to the following statement:

‘Districts receive cases from sources that include results from the clinicians in the district and local laboratories as well as new daily cases forwarded from the province’. (Guiding document for COVID-19 Surveillance Information Flow, version September 2020)

#### Surveillance data mapping

Patient level, COVID-19 test and confirmed case data were the primary COVID-19 surveillance datasets. These surveillance data were reported from the national level to subnational levels via the non-traditional information flow. The COVID-19 test and confirmed case data were sourced from public and private sector laboratories and consisted of prescribed minimum data elements recorded at specimen collection and required for COVID-19 testing. The COVID-19 data recorded at community and health facility levels were reported upward to provincial then national level (traditional information flow). Data such as close contact tracing data collected from contact tracing teams, hospitalisation data collected through a sentinel hospital surveillance system (DATCOV), COVID-19 occupational health and COVID-19 outcome data were reported through traditional information flow as COVID-19 sub-datasets. The COVID-19 sub-datasets were reported to the provincial level via different vertical reporting channels with limited to nil integration. Limited administrative and organisational integration at local levels was evidenced by the reporting of standalone COVID-19 sub-datasets that were not collated into one dataset for upward reporting. For example, COVID-19 occupational health data and COVID-19 mortality data, particularly for non-hospitalised COVID-19 deaths, were reported and available at the provincial level as distinct datasets, separate from the COVID-19 confirmed case data. A hybrid of electronic and paper-based reporting formats was identified at lower reporting levels. Paper-based reports were primarily contact tracing data captured at local levels and reported to the provincial level as aggregate contact tracing reports. At the provincial level, save for aggregate contact tracing reports, patient-level Microsoft Excel-based datasets, including the primary COVID-19 datasets (COVID-19 test and confirmed case datasets) and COVID-19 sub-datasets, predominated. Table 2 summarises the types and sources of Eastern Cape COVID-19 surveillance data determined from the document review.

**TABLE 2:** Mapping type and source of COVID-19 surveillance data; Eastern Cape, 2020 – 2021.

COVID-19 dataset or sub-dataset	Description of dataset (at provincial level)	Dataset sources	Primary information flow
**Laboratory** *Test and case data*	Patient level *SARS COV-2 tests & COVID-19 confirmed cases* *Exposure and risk factor data*	NICD & Laboratories *Electronic, centralised national-level database* *Routine surveillance system*	Non-traditional
**Contact tracing**	Aggregate *Close contact*	District reports *Contact tracing application, line lists, aggregate reports*	Traditional
**Hospitalisation**	Patient level *COVID-19 hospitalisations*	DATCOV *Sentinel hospital surveillance system for COVID-19*	Traditional
**Occupational health**	Patient level and aggregate *COVID-19 infection among health workforce*	District reports and datasets *Occupational health surveillance*	Traditional
**Outcome**	Patient level *COVID-19 mortality* *COVID-19 recoveries*	District-specific datasets & DATCOV *Line lists*	Traditional

NICD, National Institute for Communicable Diseases; COVID-19, coronavirus disease 2019.

#### Use of COVID-19 surveillance data

The document review established that surveillance data were primarily data for action, used for index case tracking and management, and close contact listing, tracking and management. Health officials reviewed surveillance data and identified clustering of cases based on person, place and time. Such reviews flagged possible outbreaks and highlighted cases from special populations or contexts, including congregate settings such as prisons, educational institutions and retirement homes. This augmented district COVID-19 response team efforts by providing early warning alerts of COVID-19 cases and settings requiring rapid verification, investigation and response. Coronavirus disease 2019 surveillance data informed the provincial response strategy; for example, it provided the rationale for prioritising available technical support and resources to Eastern Cape districts.

#### Provincial COVID-19 surveillance process: Strengths and shortcomings

This review established that a provincial surveillance process was in place and that the process, at the district level, included local surveillance networks, which availed close to real-time data for public health response. The document review established that local surveillance networks, which included private healthcare providers and non-health sector stakeholders such as the local municipality authority, had existed before COVID-19 and was involved in district-level surveillance via district Outbreak Response Teams.

Surveillance process gaps, which included data quality limitations, were identified. Missing patients’ address and contact detail data hampered the geolocation of case and test data, as exemplified by reporting unassigned cases and unallocated tests in COVID-19 epidemiological reports. For example, the proportion of COVID-19 confirmed cases not assigned to a district increased from 0.4% (338/89 853) reported on the 04th of October 2020 to 0.6% (1 044/188 893) reported on the 21st of January 2021. During the same period, the proportion of unallocated tests increased from 15% to 22%. A second shortcoming was data disharmony between provincial and district levels, as highlighted in provincial COVID-19 surveillance text, which stated that:

‘Provincial Data Points are collated from daily case line lists extracted on the online web platform. Districts may have detected cases that do not reflect on the Provincial Data Points. Such cases may be yet to reflect on the NMCSS Online Web Platform or may be cases allocated to another province due to data quality limitations.’ (Guiding document for COVID-19 Surveillance Information Flow, version September 2020)

The surveillance text reviewed alluded to data consistency challenges resulting from day-to-day data disharmony between the provincial level, which utilised centralised, non-traditional information flow introduced for COVID-19 reporting, and the district levels, which reported data availed from local surveillance networks and the provincial level. As alluded to in the quoted text, the time lag between COVID-19 case data available at the provincial level (reported via non-traditional information flow) compared to COVID-19 case data available at lower levels (reported from both traditional and non-traditional information flow) as well as data completeness challenges contributed to data disharmony between intermediate reporting levels. Routine data harmonisation meetings were held to monitor and manage data disharmony between the provincial and district reporting levels. The strengths and shortcomings established by document review of the provincial COVID-19 surveillance are summarised in Table 3.

**TABLE 3:** A summary of strengths and shortcomings established by a document review of COVID-19 surveillance, arranged by thematic area, Eastern Cape 2020–2021.

Theme	Strength	Shortcoming
Provincial COVID-19 process	Local surveillance networks established before COVID-19.	Data quality: missing geo-location data.
		Data disharmony between provincial and local level.
Technical resources for surveillance	Local availability of COVID-19 surveillance guidelines, specimen collection guides.	Surveillance approach and tools were revised and new approaches introduced not preceded by adequate training and support.
	Province-specific surveillance guiding documents developed to inform COVID-19 confirmed case and outcome reporting.	Context-specific guidelines available at provincial level and not disseminated to local levels.
Human resources for surveillance	Deployment of surge teams, including surveillance cadres, at provincial and district level to strengthen surveillance capacity and COVID-19 response.	District-level multi-discipline response teams did not always include epidemiology and surveillance cadres.
	Provincial and district teams described and tracked local epidemiology of COVID-19 and disseminated epidemiological reports.	
	Surveillance teams investigated and responded to COVID-19 outbreaks in various settings: community-level, congregate settings and among vulnerable populations.	
Technological resources for surveillance	Availability and access to national-level, web-based, centralised COVID-19 test data system.	National level, web-based COVID-19 system was not accessible at district and lower health, thus data management gaps persisted at lower levels.
	Availability and use of the NMCSS for enhanced COVID-19 surveillance.	Under-utilisation of routine public health surveillance system for enhanced COVID-19 surveillance limited availability of COVID-19 exposure and risk factor information.
	District-specific development and use of a COVID-19 close contact tracing and data management application for COVID-19 contact tracing and management.	Automated reports and dashboards were not standard across all reporting levels in the province.
	Availability and use of COVID-19 dashboards and automated COVID-19 reports strengthened and streamlined COVID-19 analysis.	Poor internet connectivity, especially in rural districts an obstacle for implementation and use of technological innovations for surveillance.

### Technical resources for surveillance

The review identified technical resources as an essential component of disease surveillance. Provincial COVID-19 preparedness efforts were bolstered by the availability of national COVID-19 technical resources (NICD 2023b). The COVID-19 technical resources, including surveillance guidelines and specimen collection guides, were disseminated at provincial, district and health facility levels and were available for reference or download from public health websites. The COVID-19 preparedness training, conducted before the first case was reported, provided for a standardised COVID-19 surveillance approach early on during COVID-19 response.

Province-specific guiding documents were developed to inform and standardise evolving COVID-19 surveillance, particularly confirmed case and outcome data reporting and management. However, the context-specific guidelines were not formally endorsed by the provincial health authority and, therefore, not distributed to district and lower reporting levels. The content of province-specific guiding documents were disseminated during training sessions, COVID-19 outbreak review meetings, data harmonisation sessions, and in response to data queries. We established that extensive preparedness training had been conducted before the province reported the first case. This training covered surveillance approaches and tools such as the Person Under Investigation (PUI) form introduced early on during COVID-19 response. Furthermore, in response to the evolving COVID-19 outbreak, surveillance approaches and tools were revised; however, adequate support and training did not always precede the revisions or new introductions.

This finding was echoed in the IAR which reported that:

‘Frequent changes in data management tools (from the PUI to specimen form) with less information for tracers. Standard Operating Procedures, templates and data collection tools were issued without users being trained.’ (IAR Report; Eastern Cape, 2021)

### Human resources for surveillance

Human resources for surveillance were a critical aspect for provincial COVID-19 surveillance and response. The provincial COVID-19 surveillance team coordinated COVID-19 surveillance activities and described and tracked the epidemiology of COVID-19 in the province. District-level multidisciplinary response teams that were constituted described and tracked the local epidemiology of COVID-19. District response teams did not always have epidemiology or surveillance cadres, as at the district level, this function was described as being historically under-resourced. The COVID-19 surge teams comprising epidemiologists, surveillance officers and biostatisticians were deployed to augment provincial and district-level COVID-19 response teams. The deployed surveillance and epidemiology officials were integrated into provincial and district COVID-19 response teams and, together with local health officials, made up the surveillance and epidemiology component of the COVID-19 response. The province and district teams were involved in various surveillance functions beyond collating, analysing and disseminating local COVID-19 statistics. From outbreak reports compiled during the review period, the provincial and district teams investigated and responded to COVID-19 outbreaks, including community-level outbreaks, some of which were related to funerals; outbreaks in educational settings, outbreaks among vulnerable populations such as those within correctional service settings, frail care and retirement homes.

### Technological resources for surveillance

Technological resources for surveillance were identified as the fourth theme. Strengths identified included the establishment of a web-based, centralised COVID-19 test data system at a national level and provincial access to the data system. The document review established that, during the review period, there were changes in how COVID-19 test and COVID-19 confirmed case data were relayed between reporting levels. Non-traditional information flow between the national and the provincial levels transitioned from emailing password-protected Microsoft Excel case line lists to provinces extracting COVID-19 test and confirmed case data from the access-controlled, web-based, national-level COVID-19 data system. This national level, web-based COVID-19 data system was, however, not accessible at district and lower health reporting levels, as the system was not decentralised and district and lower health reporting levels were not granted access rights; thus, COVID-19 data management gaps persisted at the intermediate and local health reporting levels. Another strength identified was the availability and use of the NMCSS for enhanced COVID-19 surveillance. However, underutilisation of the routine public health surveillance system for COVID-19 surveillance, coupled with inadequate follow-up training and support following the shift from the PUI form, limited the availability of COVID-19 exposure and risk factor information. A laboratory specimen submission form and the PUI form were the primary case recording tools introduced during the COVID-19 preparedness training conducted in March 2020. This PUI form was replaced by reporting of COVID-19 surveillance data via the NMCSS in May 2020. The change from paper-based PUI form to enhanced electronic reporting was not accompanied by adequate training and support. In addition, where web-based surveillance solutions were introduced, poor internet connectivity, especially in rural districts, compounded the implementation and utilisation of technological innovations. This finding was established in the IAR report, which stated:

‘Poor connectivity in the province – data capturing still done manually not electronically, leading to delays in decision making for program management and gaps in data for reporting.’ (IAR Report, Eastern Cape, 2021)

The review identified that the development and availability of COVID-19 dashboards and automated COVID-19 reports augmented and streamlined COVID-19 analysis. However, automated reports and dashboards were only standard across some reporting levels in the province. District-specific technological innovations included developing and using a web-based COVID-19 close contact tracing and data management application, which was also a best practice identified during the provincial IAR. This COVID-19 close contact data management application facilitated tracking and monitoring of COVID-19 close contacts and availed person-level contact tracing data for COVID-19 reporting.

## Discussion

The document review described the local availability of close to real-time COVID-19 data, which facilitated local public health response, especially during the containment stage of the pandemic. Availability of close to real-time data was enabled, in part, by local surveillance networks that had been in existence before the COVID-19 pandemic and been involved in local-level surveillance and response activities. This prior collaboration enabled local surveillance networks to respond to local surveillance data needs created by the rapidly evolving and previously undescribed public health emergency.

Data incompleteness, which was a finding of the document review, was not unique to the provincial context and has been described for COVID-19 data reported in other contexts (Costa-Santos et al. 2021; Gold et al. 2021; Talisuna et al. 2022). For geolocation data, one study described the completeness of postal address code and geographic area of residential address data at above 89% (Vieira Ribas et al. 2022). Another study described a decrease in the completeness of place of residence data from 71% in epidemiological week three of 2020 to less than 2% for epidemiological weeks seven to nine of 2020 (Ricks et al. 2022). Although our document review did not assess the degree of completeness nor factors related to COVID-19 data completeness, it established the occurrence of unassigned or misallocated COVID-19 cases resulting from missing or incomplete geolocation variables such as residential address and telephone numbers, which delayed and, in some instances, prevented assignment of COVID-19 case data to response teams. Implications of unassigned or misallocated COVID-19 cases include nil respective public health response to the case, which would have dire consequences during the containment phase of the response. A lack of public health response because of poor data quality warrants focussed considerations to promote the recording and collection of good quality geolocated data that enable timely and complete public health response.

Delayed or incorrect geolocation of COVID-19 case data at national and provincial levels contributed to data disharmony between intermediate reporting levels, wherein district-level reflected, almost real-time COVID-19 cases, some of which were either still unassigned or misallocated to another province and thus not reported at the provincial level. Time lag in reporting and processing not only influenced COVID-19 data availability and use (Ricks et al. 2022) at provincial and district levels, but contributed to data disharmony between data reported at local versus provincial and national level because of different reporting cut-off times between centralised national reporting and local reporting at health facility and community level. The COVID-19 data at the provincial level was predominantly from non-traditional information system, while district-level data was sourced from both traditional hierarchical and non-traditional information systems. District-level reporting would have accounted for real-time reflection of COVID-19 case burden and a real-time indication of resources utilised and resources required, whereas provincial reports reflected official COVID-19 statistics collated and reported at the national level. Dual COVID-19 information pathways in the absence of a provincial, web-based, integrated COVID-19 data management system compounded data disharmony introduced by data incompleteness. A local, web-based integrated surveillance system would have enabled coordinated reporting and management of case data between local, district and provincial reporting levels.

There are several implications of the data quality findings of this document review. Firstly, poor data quality affects the validity and credibility of data, which impacts the usefulness of surveillance data to guide decision-making and direct public health interventions (Gold et al. 2021; Groseclose & Buckeridge 2017). Secondly, public health messaging and communication, which fosters trust and promotes cooperation and compliance (Adebisi et al. 2021; Judson et al. 2022; Sagan et al. 2021), requires good-quality surveillance data. Therefore, poor data quality, including data inconsistency, could undermine continued public trust in the local health authority and render health authority response efforts ineffective.

The review determined the existence of dual information systems that collected COVID-19 sub-datasets via vertical reporting channels. Routine public health surveillance in the province follows the traditional hierarchical flow of information from the diagnosing healthcare worker into the NMCSS and upwards to multiple stakeholders at different health reporting levels. The traditional information pathway is provided for by the notifiable medical conditions regulations (NDoH 2017). The electronic reporting platform of the NMCSS allows multi-level, real-time relay of information from the reporting source (NICD 2021); however, this information flow maintains the traditional information flow. The COVID-19 surveillance data were relayed via multiple vertical reporting channels with information flowing via the dual pathways of the traditional and non-traditional information system.

This finding has several considerations; firstly, vertical reporting channels resulted in the recording and reporting similar or related COVID-19 data multiple times on different vertical reporting platforms. A study in Italy described how the lack of integration of COVID-19 surveillance contributed to data entry errors and missing data emanating from recording similar data several times on multiple reporting tools (Costa-Santos et al. 2021). Secondly, the collection of numerous sub-datasets combined with limited integration of COVID-19 reporting could be an additional factor for data incompleteness established by this review. Thirdly, on further reflection, multiple vertical reporting channels may have collected inconsistent variables across COVID-19 sub-datasets. Inconsistencies such as missing critical variables to link COVID-19 sub-datasets, would have further limited collation and integration of COVID-19 sub-datasets at the provincial level. Lastly, implications for collecting multiple and similar data on the health workforce should be explored, as human resources for surveillance are an essential enabler for good quality surveillance data. Additional reporting systems would have increased the public health surveillance burden on a strained health workforce. This extra data collection and reporting burden on data providers would have further, negatively impacted the quality of surveillance data collected (Costa-Santos et al. 2021). Gold and co-authors reported how electronic reporting and associated automated workflows reduce the reporting burden on data providers by replacing manual reporting processes, which in turn increases reporting timeliness (Gold et al. 2021).

Even though a centralised web-based COVID-19 test data system was available at the national level, it firstly was not accessible to lower health reporting levels (Silal et al. 2022), and secondly, did not incorporate COVID-19 sub-datasets such as occupational health and outcome data (Silal et al. 2022). Thus, there was a need for a web-based, integrated COVID-19 data management system accessible at all health reporting levels within the province to improve data gaps such as data disharmony, data incompleteness and multiple COVID-19 surveillance datasets managed independently. A study set in Portugal argues for a single centralised, robust surveillance system that collects and collates required surveillance data from various platforms and registries while meeting the information needs of diverse stakeholders (Ricoca Peixoto et al. 2023). Such a system should cater to the different public health data generated and required during the different phases of the emergency management cycle (Judson et al. 2022; Ricoca Peixoto et al. 2023). A study that reviewed IAR reports of 18 African countries echoes the need for robust integrated surveillance systems. The study also described the role of the IDSR strategy in COVID-19 surveillance (Talisuna et al. 2022), which provides considerations for establishing integrated, flexible public health surveillance in our context.

The availability and use of the routine public health surveillance system, the NMCSS, for enhanced COVID-19 surveillance was an opportunity that was sub-optimally utilised for COVID-19 surveillance in the province. Enhanced COVID-19 data were initially collected and captured centrally on a REDCap platform and subsequently collected at a local level via the NMCSS. Underutilisation of the NMCSS for COVID-19 surveillance, established by this document review, could have resulted from introducing the PUI form during COVID-19 preparedness training to a captive health workforce audience anticipating response to a novel respiratory virus. Secondly, the change in COVID-19 reporting from the REDCap-based PUI form system to the NMCSS is described as having needed more follow-up training and support. This finding suggests subpar introduction and training on the revised surveillance approach. We hypothesise that introducing the PUI form to a captive audience, subpar introduction and training of revised surveillance approaches, and poor internet connectivity contributed to sub-optimal utilisation of the NMCSS for COVID-19 surveillance. A review of COVID-19 surveillance highlighted how pandemics can disrupt surveillance systems, resulting in changes in the use, availability and completeness of surveillance data (Ricoca Peixoto et al. 2023), provides further argument for robust routine surveillance systems.

The research finding of transitioning COVID-19 surveillance to an existing, routine, national surveillance platform and the finding of sub-optimal availability of both clinical notification and epidemiological investigation data throughout the COVID-19 pandemic is described by other studies (Gold et al. 2021; Ricoca Peixoto et al. 2023). The findings emphasise the importance of developing and strengthening existing routine surveillance systems that can be leveraged for surveillance during high burden or unexpected public health events. However, it is worth observing that increasing case burden may lead to incomplete reporting, as determined by a review of global COVID-19 reporting, which described a decline in the proportion of cases with COVID-19 exposure information, as the pandemic progressed. For example, one study reported that the proportion of cases reported with exposure data decreased from 100% in epidemiological week three to 87% in week six and 2% in week nine (Ricks et al. 2022).

Groseclose and Buckridge (2017) emphasise the importance of public health surveillance systems that are flexible and adaptable to changing disease epidemiology, clinical practice, evolving information needs, and available technologies. Although the document review identified the need for a robust, integrated, web-based surveillance system, several studies recognise that unprecedented public health events could necessitate additional surveillance capacity (Adebisi et al. 2021; Judson et al. 2022). As such, the emergence of additional surveillance systems, such as DATCOV and the introduction of the non-traditional information flow for COVID-19 with its web-based, centralised COVID-19 test data system is not unexpected, as public health emergencies may prompt the development of multiple, diverse or complementary reporting systems (Adebisi et al. 2021; Judson et al. 2022; Khamis Ibrahim 2020; Kinkade et al. 2022). Although ad hoc surveillance systems may be necessary, the implications and impacts of introducing ad hoc surveillance systems must be anticipated and mitigated. Harnessing existing technologies and digital infrastructure to rapidly respond to public health emergencies was a key lesson from the information and communication response to the 2014 Ebola Virus Disease outbreak (Kinkade et al. 2022). Heeding lessons from the outbreak, Sierra Leone, Sri Lanka, and Uganda adapted their existing information systems to meet COVID-19 surveillance needs. These countries did not establish additional parallel systems for COVID-19 surveillance as they leveraged previous investments into national health information reporting systems (Kinkade et al. 2022). Such countries argue for developing robust but flexible routine surveillance systems that will not necessitate parallel or complementary reporting systems during public health emergencies.

When complementary data reporting pathways are established, they must be interoperable and linked to the routine public health surveillance system (Judson et al. 2022; Richards et al. 2017). As the public health response de-escalated, COVID-19 surveillance had to be integrated into routine public health surveillance. A ministerial advisory on integrating COVID-19 activities into routine healthcare services provided the rationale for transitioning from acute pandemic response to a sustainable COVID-19 prevention, detection and management approach (Ministerial Advisory Committee on COVID-19 2022). Thus, within the public health surveillance context, should future public health emergencies necessitate complementary surveillance systems, the interoperability and linkability of such systems is essential for integrating complementary surveillance capacities into the routine surveillance systems. A systematic review of IDSR implementation in Africa highlighted the importance of streamlining the surveillance process and building on existing capacity and infrastructure as a critical consideration for developing robust public health surveillance systems (Wolfe et al. 2021).

Investing in quality digital and/or electronic data management systems is pertinent for effective public health surveillance and the detection of and response to public health events (Adebisi et al. 2021; Richards et al. 2017). This document review identified the need for broader application of technological innovations for surveillance (Talisuna et al. 2022); however, obstacles exist to the practical application of such innovations. Described obstacles include poor internet connectivity in some regions of the predominantly rural province (Statistics South Africa 2018). Barriers to using digital surveillance tools and contact tracing applications for COVID-19 surveillance in rural communities in Africa include limited access to smartphones and limited to nil access to electricity (Adebisi et al. 2021). Understanding the hindrances to the use of technological innovations for surveillance in the Eastern Cape will assist in resolving the limited application of technological innovations for surveillance described by the document review.

Human resources for surveillance was a gap identified and managed through rationalising available human resources and deploying surge staff. The deployment of field epidemiologists to support data collation, analysis, reporting, and COVID-19 cluster detection was reported in several African countries (Talisuna et al. 2022), which alludes to broader human resources for surveillance gaps beyond the provincial context. Surge team deployment was a short-term strategy that augmented district-level response teams that would otherwise not include surveillance or epidemiology cadres. Post-COVID-19, the district surveillance cadre gap persists. In addition to this, the technical epidemiological skills gained during the COVID-19 response may not be sustained or carried over beyond COVID-19 response activities, as officials deployed to the district and local reporting levels have most likely since returned to their routine, and sometimes ‘non-surveillance’ roles.

The human resources for surveillance gap is a long-standing challenge in Africa (Adebisi et al. 2021; Nsubunga et al. 2010; Wolfe et al. 2021). Robust and agile national public health surveillance and response require a well-resourced, competent public health cadre at national and sub-national levels (Nsubunga et al. 2010). Surveillance expertise is needed to analyse data and generate information for public health response and intelligence for decision-making (Ricks et al. 2022). This document review also established that shortage of skilled surveillance staff contributed to poor surveillance data management and sub-optimal transmission and use of surveillance data (Adebisi et al. 2021; Costa-Santos et al. 2021). Local-level epidemiology needs can be partly managed by applying technological innovations to streamline the collection and processing of data and thus reduce the surveillance burden on the health workforce (Gold et al. 2021; Richards et al. 2017). Such innovations include integrating the collection of surveillance data and automating the processing, analysis and reporting of surveillance data.

Although COVID-19 preparedness training was conducted, key COVID-19 surveillance stakeholders reflected inadequate follow-up support for evolving surveillance processes and tools. The transition from the PUI form to the enhanced COVID-19 NMCSS described by Silal and co-authors (Silal et al. 2022) highlights evolving surveillance processes that would have required ongoing training and support. This support gap could be linked to province-specific guiding documents, which were developed to inform on the evolving COVID-19 surveillance approach but were unavailable for reference at lower reporting levels, as they had not been disseminated further. Notwithstanding that the content of the context-specific guidelines was disseminated through various means, nil distribution of province-specific surveillance guidelines to lower reporting levels and, thus, their unavailability for reference at the local level is a critical surveillance gap. An assessment of COVID-19 response reviews of some African countries described delays in the development and dissemination, review and update of guidelines (Talisuna et al. 2022). Surveillance system enablers include information technology that supports data collection, collation, analysis and dissemination and the availability of systems and directories for disseminating public health alerts, bulletins and guidelines (Groseclose & Buckeridge 2017). Such systems and directories provide a standardised framework for distributing relevant technical resources at sub-national levels, which can be used when disseminating public health resources.

Although the qualitative review of the COVID-19 surveillance documents identified important strengths and opportunities to strengthen public health surveillance in the province, some limitations should be considered. This desktop review was limited to information available at the provincial level at the time of the review. In spite of the fact that the document review incorporated the inputs of key surveillance stakeholders collated during the provincial COVID-19 IAR, it represents a limited scope of the provincial COVID-19 surveillance approach; for example, it did not assess COVID-19-related contact tracing. An in-depth evaluation of public health surveillance, including at district, health facility and community levels, may highlight additional aspects beyond those established by the document review. Despite being limited in scope, the document review provides critical findings for strengthening routine public health surveillance.

## Recommendations

Based on the findings of the document review, several recommendations are proposed to strengthen routine public health surveillance in the province and beyond. The document review established that data quality was a shortcoming of provincial COVID-19 surveillance; hence, the first recommendation is to implement strategies that improve the recording of geolocation data collected in the community. Collection of geolocation data will improve data completeness for data collected in rural communities and informal settlements. Recording geolocation data, can be achieved through the use of technology that captures geographic coordinate system data.

Automated COVID-19 reports and COVID-19 dashboards facilitated COVID-19 data analysis and information dissemination. We recommend that such data processing and visualisation approaches be applied beyond COVID-19. Capacitating local officials and providing ongoing support and mentorship in surveillance data management will assist in sustaining skills gained beyond COVID-19.

The document review established the need for a flexible, integrated, web-based data management system. On this basis, the second recommendation is for national and provincial health authorities to advance the existing routine public health surveillance systems so they can be rapidly and efficiently adapted for surveillance during diverse and unprecedented public health events. Central to this, we recommend a comprehensive review of public health surveillance gaps and factors that necessitated the development of parallel and ad hoc reporting systems. The findings of the proposed review should be incorporated into efforts to strengthen the existing routine public health surveillance system.

Under-resourcing of the surveillance function at the district level and the stop-gap measure that will not enable sustained local capacity development were described. In addition, poor Internet connectivity in rural districts hampered technological innovations that could have addressed human resources for surveillance constraints. To address the resource-related constraints, we recommend investment in human resources for surveillance and investment in information and communication technology infrastructure for surveillance. Such investments are critical to developing robust, agile, integrated, routine surveillance systems. This recommendation is proposed while realising that engagements and investments may require stakeholders beyond the provincial health authority.

## Conclusion

This article provides a sub-national perspective of COVID-19 surveillance and articulates and archives reflections on the strengths and shortcomings of the provincial COVID-19 surveillance process. It escalates considerations needed to strengthen routine public health surveillance: a critical public health preparedness strategy. The findings provide a basis to initiate broader engagements on the recommendations for developing robust, integrated public health surveillance systems. Routine public health surveillance systems require investment and development to collect and process enhanced and evolving surveillance data and provide good quality data for public health response and decision-making.

## References

[CIT0001] Adebisi, Y.A., Rabe, A. & Lucero-Prisno Iii, E., 2021, ‘COVID-19 surveillance systems in 13 African countries’, *Health Promotion Perspectives* 2021(4), 382–392. 10.34172/hpp.2021.49 PMC8767077 35079582

[CIT0002] Arvisais-Anhalt, S., Lehmann, C.U., Park, J.Y, Araj, E., Holcomb, M., Jamieson, A.R. et al., 2021, ‘What the Coronavirus Disease 2019 (COVID-19) pandemic has reinforced: The need for accurate data’, *Clinical Infectious Diseases* 72(6), 920–923. 10.1093/cid/ciaa1686 33146707 PMC7665390

[CIT0003] Bowen, G.A., 2009, ‘Document analysis as a qualitative research method’, *Qualitative Research Journal* 9(2), 27–40. 10.3316/QRJ0902027

[CIT0004] Costa-Santos, C., Luisa Neves, A., Correia, R., Santos, P., Monteiro-Soares, M., Freitas, A. et al., 2021, ‘COVID-19 surveillance data quality issues: A national consecutive case series’, *BMJ Open* 11(12), 47623. 10.1136/bmjopen-2020-047623 PMC8649880 34872992

[CIT0005] Gold, J.A.W., DeCuir, J., Coyle, J.P., Duca, J.P., Adjemian, J., Anderson, K.N. et al., 2021, ‘COVID-19 case surveillance: Trends in person-level case data completeness, United States, April 5–September 30, 2020’, *Public Health Reports* 136(4), 466. 10.1177/00333549211006973 33789540 PMC8203037

[CIT0006] Groseclose, S.L. & Buckeridge, D.L., 2017, ‘Public health surveillance systems: Recent advances in their use and evaluation’, *Annual Review of Public Health* 38(1), 57–79. 10.1146/annurev-publhealth-031816-044348 27992726

[CIT0007] IFRCS, 2020, *World disasters report 2020*, Geneva, viewed 30 June 2022, from https://www.ifrc.org/document/world-disasters-report-2020.

[CIT0008] Jassat, W., Mudara, C., Ozougwu, L. et al., 2021, ‘Increased mortality among individuals hospitalised with COVID-19 during the second wave in South Africa’, *medRxiv*. 10.1101/2021.03.09.21253184

[CIT0009] Judson, S.D., Torimiro, J., Pigott, D.M., Maima, A., Mostafa, A., Samy, A. et al., 2022, ‘COVID-19 data reporting systems in Africa reveal insights for future pandemics’, *Epidemiology and Infection* 150, e119. 10.1017/S0950268822001054 35708156 PMC9237488

[CIT0010] Khamis Ibrahim, N., 2020, ‘Epidemiologic surveillance for controlling Covid-19 pandemic: Types, challenges and implications’, *Journal of Infection and Public Health* 13(11), 1630–1638. 10.1016/j.jiph.2020.07.019 32855090 PMC7441991

[CIT0011] Kinkade, C., Russpatrick, S., Potter, R., Saebo, J., Sloan, M., Odongo, G. et al., 2022, ‘Extending and strengthening routine DHIS2 surveillance systems for COVID-19 responses in Sierra Leone, Sri Lanka, and Uganda’, *Emerging Infectious Diseases* 28(13), S42–S48. 10.3201/eid2813.220711 36502427 PMC9745217

[CIT0012] Ministerial Advisory Committee on COVID-19, 2022, *Integrating COVID-19 diagnosis, prevention and care into routine healthcare services*, viewed 03 July 2024, from https://sacoronavirus.co.za/wp-content/uploads/2022/06/MAC-on-COVID-19_Advisory-on-Integrating-COVID-19-into-Routine-Healthcare-Services_8June2022.pdf.

[CIT0013] Morgan, O.W., Aguilera, X., Ammon, A., Amuasi, J., Fall, I.S., Frieden, T. et al., 2021, ‘Disease surveillance for the COVID-19 era: Time for bold changes’, *The Lancet* 397(10292), 2317–2319. 10.1016/s0140-6736(21)01096-5 PMC8121493 34000258

[CIT0014] NDoH, 2017, *Regulations relating to the surveillance and the control of notifiable medical conditions*, National Department of Health, Pretoria, viewed 18 May 2021, from https://www.gov.za/sites/default/files/gcis_document/201712/41330gon1434.pdf.

[CIT0015] NICD, 2021, *Notifiable medical conditions surveillance system*, viewed 30 November 2021, from https://www.nicd.ac.za/nmc-overview/overview/.

[CIT0016] NICD, 2022, *NICD National COVID-19 hospital surveillance – Friday 30 December 2023*, National Institute for Communicable Diseases, Johannesburg, viewed 04 May 2024, from https://www.nicd.ac.za/wp-content/uploads/2023/01/NICD-COVID-19-Daily-Sentinel-Hospital-Surveillance-report-National-20221230.pdf.

[CIT0017] NICD, 2023a, *COVID-19 weekly epidemiology brief: Week ending 7 January 2023 (Week 1 of 2023)*, National Institute for Communicable Diseases, Johannesburg, viewed 17 April 2023, from https://www.nicd.ac.za/wp-content/uploads/2023/01/COVID-19-Weekly-Epidemiology-Brief-week-1-2023-.pdf.

[CIT0018] NICD, 2023b, *Diseases A to Z index | COVID-19*, viewed 21 July 2023, from https://www.nicd.ac.za/diseases-a-z-index/disease-index-covid-19/.

[CIT0019] Nsubuga, P., White, M.E., Thacker, S.B., Anderson, M.A., Blount, S.B., Broome, C.V. et al., 2006, ‘Public health surveillance: A tool for targeting and monitoring tnterventions’, in D.T. Jamison, J.G. Breman, A.R. Measham AR, et al. (eds.), *Disease control priorities in developing countries*, 2nd edn., pp. 997–1015, Oxford University Press, Washington DC, viewed 18 April 2021, from https://www.ncbi.nlm.nih.gov/books/NBK11770/pdf/Bookshelf_NBK11770.pdf.

[CIT0020] Nsubunga, P., Nwanyanwu, O., Nkengasong, J.N., Mukanga, D. & Trostle, M., 2010, ‘Strengthening public health surveillance and response using the health systems strengthening agenda in developing countries’, *BMC Public Health* 10(suppl 1), S5. 10.1309/AJCP8GYX8KTKDATZ 21143827 PMC3005577

[CIT0021] Richards, C.L., Iademarco, M.F., Atkinson, D., Pinner, R.W., Yoon, P., Mac Kenzie, W.R. et al., 2017, ‘Advances in public health surveillance and information dissemination at the centers for disease control and prevention’, *Pubic Health Reports* 132(4), 403–410. 10.1177/0033354917709542 PMC5507423 28609194

[CIT0022] Ricks, P.M., Njie, G.J., Dawood, F.S., Blain, A.E., Winstead, A., Popoola, A. et al., 2022, ‘Lessons learned from CDC’s global COVID-19 early warning and response surveillance system’, *Emerging Infectious Diseases* 28(13), S8–S16. 10.3201/eid2813.212544 36502410 PMC9745250

[CIT0023] Ricoca Peixoto, V., Vieira, A., Aguiar, P., Sentis, A., Carvalho, C., Thomas, D.R. et al., 2023, ‘COVID-19 surveillance: Large decrease in clinical notifications and epidemiological investigation questionnaires for laboratory-confirmed cases after the 2nd epidemic wave, Portugal March 2020–July 2021’, *Frontiers in Public Health* 11, 963464. 10.3389/fpubh.2023.963464 36969655 PMC10035048

[CIT0024] Rogers, D.P., Anderson-Berry, L., Bogdanova, A-M., Fleming, G., Gitay, H., Kahandawa, S. et al., 2020, ‘COVID-19 and lessons from multi-hazard early warning systems’, *Advances in Science and Research* 17, 129–141. 10.5194/asr-17-129-2020

[CIT0025] Sagan, A., Webb, E., Azzopardi-Muscat, N., Mata, I.d.l., McKee, M. & Figueras, J., 2021, *Lessons for building back better Health Policy Health Policy Series*, Series No. 56, Health Policy Series, viewed 28 July 2023, from https://eurohealthobservatory.who.int.

[CIT0026] Saikat, S., Selbie, D., McDarby, G., Mustafa, S., Petrova, M., Seifeldin, R. et al., 2023, ‘Editorial: Health systems recovery in the context of COVID-19 and protracted conflict’, *Frontiers in Public Health* 11, 1205286. 10.3389/fpubh.2023.1205286 37293611 PMC10246765

[CIT0027] Silal, S.P., Groome, M.J., Govender, N., Pulliam, J.R.C., Ramadan, O.P., Puren, A. et al., 2022, ‘Leveraging epidemiology as a decision support tool during the COVID-19 epidemic in South Africa’, *South African Medical Journal* 112(5b), 361–365. 10.7196/SAMJ.2022.v112i5b.16061 35783465 PMC7612950

[CIT0028] Statistics South Africa, 2018, *Provincial profile: Eastern Cape community survey 2016*, Pretoria, viewed 15 February 2021, from www.statssa.gov.zainfo@statssa.gov.za.

[CIT0029] Statistics South Africa, 2022a, *General household survey 2021. 23 June*, Pretoria, viewed 06 September 2022, from www.statssa.gov.za,info@statssa.gov.za,Tel+27123108911.

[CIT0030] Statistics South Africa, 2022b, *Mid-year population estimates 2022*, Pretoria, viewed 17 May 2023, from https://www.statssa.gov.za/publications/P0302/P03022022.pdf.

[CIT0031] Talisuna, A., Iwu, C., Okeibunor, J., Stephen, M., Musa, E.O., Herring, B.L. et al., 2022, ‘Assessment of COVID-19 pandemic responses in African countries: Thematic synthesis of WHO intra-action review reports’, *BMJ Open* 12(5), 1–7. 10.1136/bmjopen-2021-056896 PMC9062458 35501083

[CIT0032] Tessema, S.K., Inzaule, S.C., Christoffels, A., Kebede, Y., Oliveira, T.d., Ogwell Ouma, A.E. et al., 2020, ‘Accelerating genomics-based surveillance for COVID-19 response in Africa’, *The Lancet Microbe* 1(6), e227–e228. 10.1016/S2666-5247(20)30117-8 32838350 PMC7434434

[CIT0033] Thacker, S.B. & Berkelman, R.L., 1988, ‘Public health surveillance in the United States’, *Epidemiologic Reviews* 10(1), 164–190. 10.1093/oxfordjournals.epirev.a036021 3066626

[CIT0034] Uwe, F., Åkerström, M., Banks, M., et al., 2014, *The SAGE handbook of qualitative data analysis*, in K. Metzier, I. Antcliff & N. Hankins (eds.), 1st edn., SAGE Publications, Los Angeles, CA, viewed 10 December 2021, from https://www.ufs.ac.za/docs/librariesprovider68/resources/methodology/uwe_flick_(ed-)-_the_sage_handbook_of_qualitative(z-lib-org)-(1).pdf?sfvrsn=db96820_2.

[CIT0035] Vieira Ribas, F., Cristina Dias Custódio, A., Vieira Toledo, L., Henriques, B.D., de Oliveira Sediyama, C.M.N. & Chagas de Freitas, B.A., 2022, ‘Completeness of notifications of severe acute respiratory syndrome at the national level and in a regional health care in the state of Minas Gerais, during the COVID-19 pandemic, 2020’, *Epidemiol Serv Saude* 31(2), e2021620. 10.1590/s1679-49742022000200004 35730813 PMC9887980

[CIT0036] WHO, 2014, *Early detection, assessment and response to acute public health events: Implementation of early warning and response with a focus on event-based surveillance*, WHO, viewed 02 November 2022, from WHO/HSE/GCR/LYO/2014.4.

[CIT0037] WHO, 2021, *Guidance for conducting a country COVID-19 intra-action review (IAR)*, viewed 10 July 2021, from https://www.who.int/publications/i/item/WHO-2019-nCoV-Country_IAR-2020.1.

[CIT0038] Wolfe, C.M., Hamblion, E.L., Dzotsi, E.K., Mboussou, F., Eckerle, I., Flahault, A. et al., 2021, ‘Systematic review of Integrated Disease Surveillance and Response (IDSR) implementation in the African region. *PLoS One* 16(2), e0245457. 10.1371/journal.pone.0245457 33630890 PMC7906422

